# Supplementary figures and images for: Reinforcement amid genetic diversity in the Candida albicans biofilm regulatory network
Source: PLoS Pathog. 2023 Jan 25;19(1):e1011109. doi: 10.1371/journal.ppat.1011109 (PMC9901766; doi:10.1371/journal.ppat.1011109)

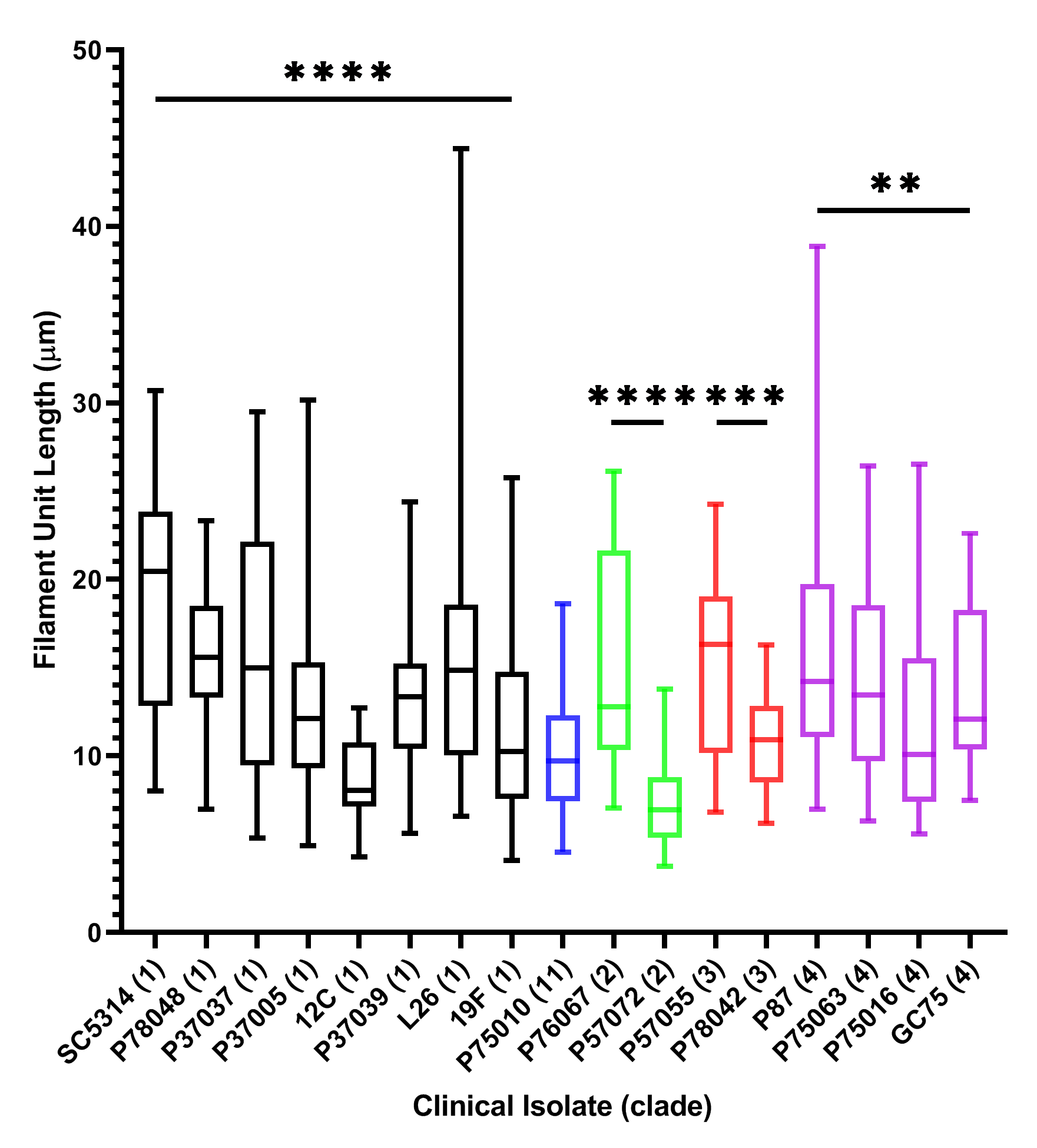

Supplement: S1 Fig — WT clinical isolate strains were assayed for planktonic hyphal formation in RPMI+10% serum at 37°C. Cultures were grown for 4 hours with 60rpm rotation. Hyphal units were measured between septa or between yeast cell and hyphal tip for 50–100 cells in 3 fields of view. Clade number is designated in parentheses. One way ANOVA was performed on Clade 1 and Clade 4 isolates and showed significant difference within each clade (p<0.0001 and p = 0.0066 respectively). Unpaired T-tests were performed within Clades 2 and 3. These indicated significant differences in filament unit length within the two clades (p<0.0001 and p = 0.0002 respectively). (TIF) [file ppat.1011109.s001.tif]

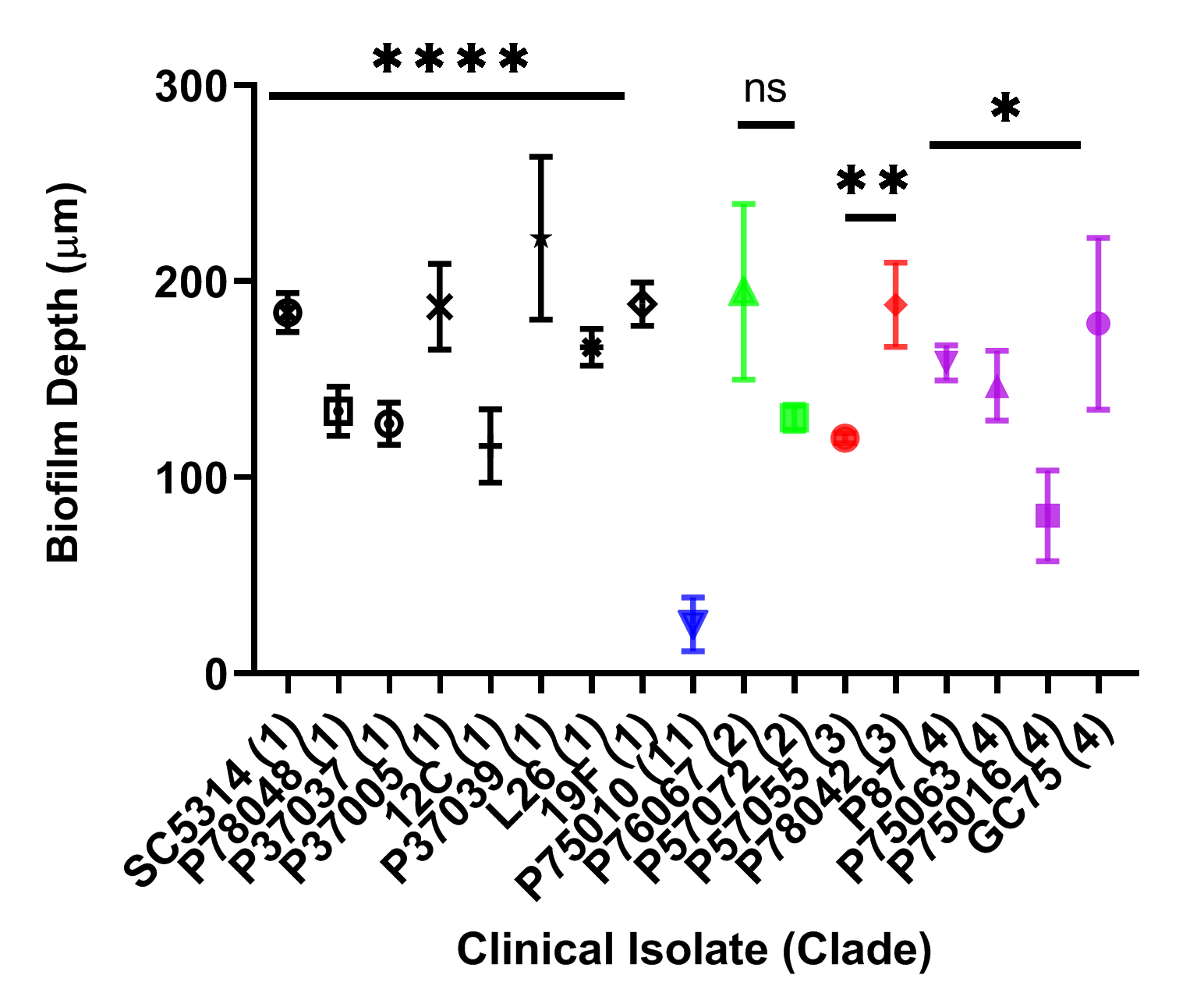

Supplement: S2 Fig — WT clinical isolate strains were assayed for biofilm formation ability in RPMI + 10% FBS at 37°C for 24hrs using the “Silicone substrate–confocal microscopy” method. Biofilm depth was measured at three different points of the biofilm for two technical replicates. Clades 1 and 4 strains were each compared via one-way ANOVA with resultant p-values of <0.0001 and 0.0101 respectively. Clades 2 and 3 biofilms were compared via unpaired T-tests. Clade 3 showed significant variation with a p-value of 0.0056 while Clade 2 isolates did not show significant variation. (TIF) [file ppat.1011109.s002.tif]

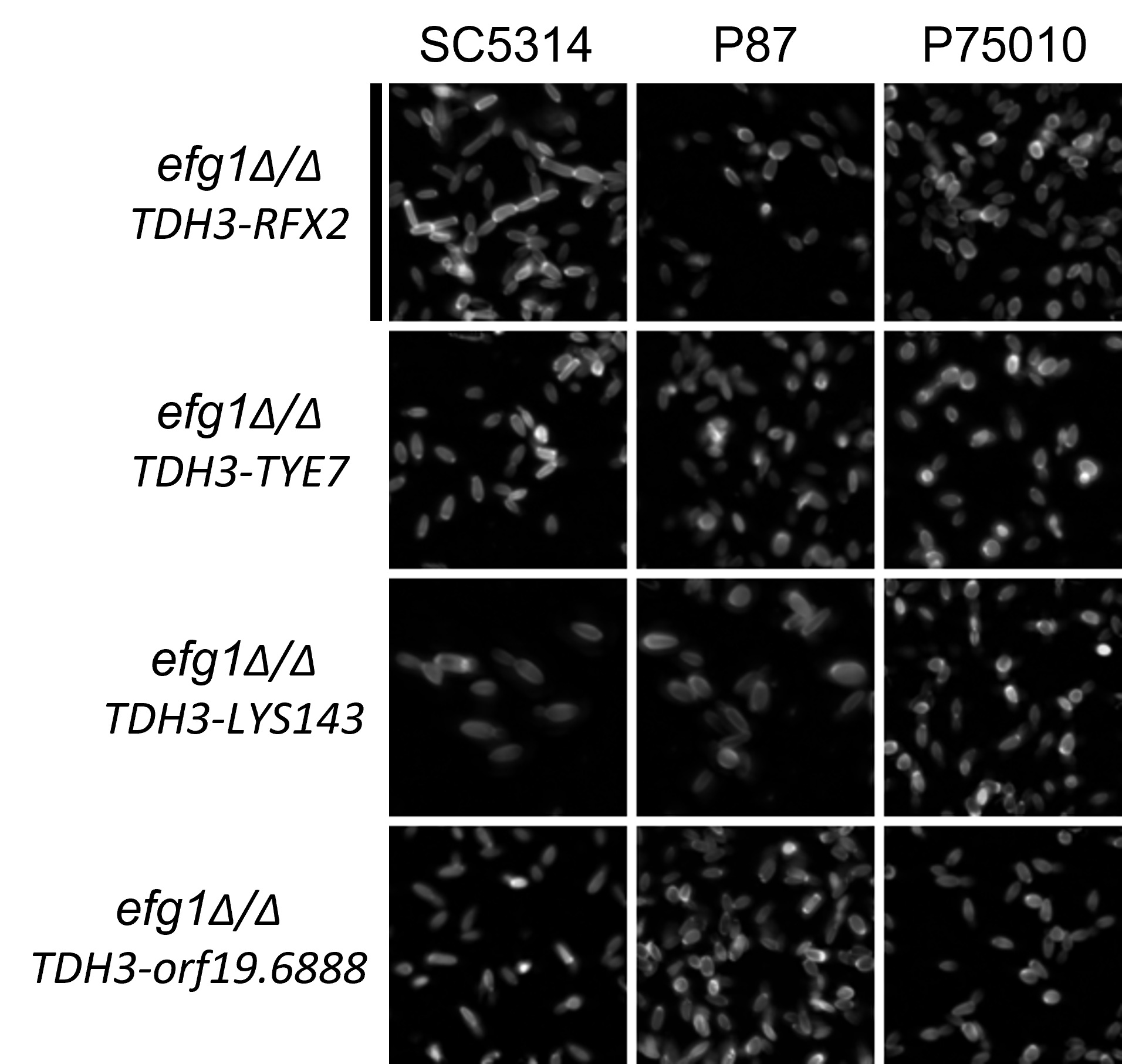

Supplement: S3 Fig — Fluorescence image of SC5314, P87, and P75010 efg1Δ/Δ TDH3-RFX2, efg1Δ/Δ TDH3-TYE7, efg1Δ/Δ TDH3-LYS143, and efg1Δ/Δ TDH3-orf19.6888 mutant planktonic hyphal formation in RPMI+10%FBS 37°C for 4hrs. Samples were fixed in 4% formaldehyde in 1X PBS and stained with Calcofluor-white. Scale bar indicates 80 μm. (TIF) [file ppat.1011109.s003.tif]

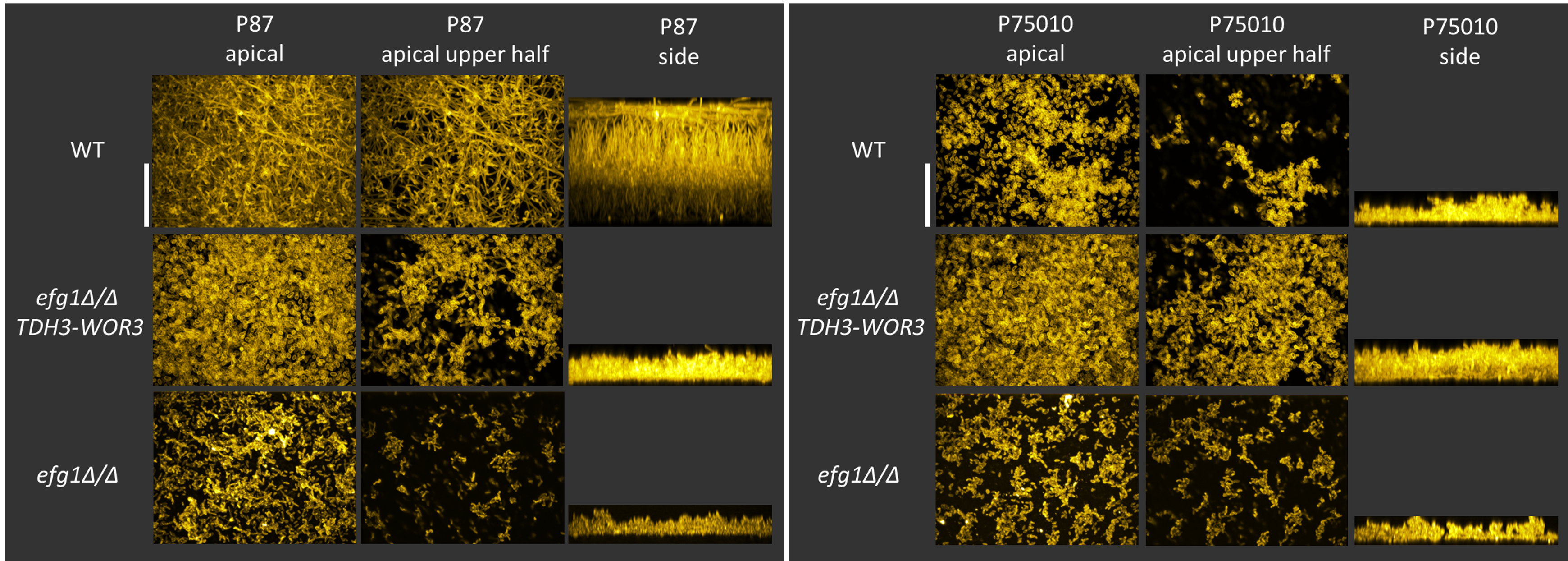

Supplement: S4 Fig — Biofilms were imaged after 24 hrs at 37°C in RPMI+10% FBS for WT, efg1Δ/Δ, and efg1Δ/Δ TDH3-WOR3 strains derived from P87 (Panel A) or P75010 (Panel B) using the “Silicone substrate–confocal microscopy” method. Maximum intensity apical projections of the entire biofilm (Left column) and the top half of the biofilm (Middle column) were generated for each strain. Side view projections (Right column) were also generated. Datasets for efg1Δ/Δ mutant images were the same as those used in Fig 2. Scale bar indicates 60 μm. (TIF) [file ppat.1011109.s004.tif]
