# Supplementary material for: Reinforcement amid genetic diversity in the Candida albicans biofilm regulatory network
Source: PLoS Pathog. 2023 Jan 25;19(1):e1011109. doi: 10.1371/journal.ppat.1011109 (PMC9901766; doi:10.1371/journal.ppat.1011109)
Supplement: Attachment — Submitted filename: Cravener et al Response to Reviews final.docx [file ppat.1011109.s011.docx]

Thank you very much for submitting your manuscript "Reinforcement amid genetic diversity in the Candida albicans biofilm regulatory network" for consideration at PLOS Pathogens. As with all papers reviewed by the journal, your manuscript was reviewed by members of the editorial board and by several independent reviewers. The reviewers appreciated the attention to an important topic. Based on the reviews, we are likely to accept this manuscript for publication, providing that you modify the manuscript according to the review recommendations.

All three reviewers acknowledge the amount and quality of the work presented in your manuscript, although they also express some concerns about the extent to which the study changes our understanding of the regulatory network controlling biofilm formation compared to current models established by previous work (including your own). Nevertheless, they agree that the study stands out by the inclusion of 17 different strains for the analysis, and they highlight the discovery of Wor3 as a new positive regulator of biofilm formation and its unexpected positive relationship with Efg1 in this program.

While you should consider all suggestions made by the reviewers to improve the clarity of the manuscript and highlight the main novel aspects, please pay specific attention to the following:

What is the main basis of strain-specific differences, as opposed to commonalities, and which role may Wor3, Brg1, and Ume6 play here (reviewer 1, part III2; reviewer 3, part IIa and c)?

We have expanded our discussion of strain differences in a few ways, though we kept this material succinct because it was not a focus of this particular study.

- lines 85-94. We include a summary of how partner TFs shape Efg1 outputs (a recap of Do et al. 2022).
- lines 285-297. We provided simple instructions for how an enterprising investigator might use our data to dissect strain differences in Efg1 outputs.
- lines 299-304. We provided a simple explanation for the relationship of Wor3, Brg1, and Ume6 to differences in hypha-associated gene expression among strains.

Please explain how overexpressed WOR3 restores biofilm formation in the absence of Efg1 without (?) forming hyphae (reviewer 1, part III.5-6).

This insightful question prompted us to conduct imaging of the *efg1*Δ/Δ *TDH3-WOR3* biofilms in three strain backgrounds. The data are included in new Figures 7 and S8 and presented in lines 217-226. These biofilms are quite rich in yeast cells overall, but we were able to find hyphal filaments by examining the upper half of the biofilm. They are clearest in apical views. The filaments were found in SC5314 and P87, but not in the weakly filamentous P75010.

Discuss how Wor3 may both antagonize (in the white-opaque switch) and support (in biofilm formation) Efg1 function (reviewer 2, part II).

We speculate that the heat shock response, which is known to govern biofilm/hyphal genes, is responsible for modifying Wor3 activity in lines 377-384. Temperature is high when Wor3 cooperates with Efg1, and low when Wor3 antagonizes Efg1.

Please prepare and submit your revised manuscript within 30 days. If you anticipate any delay, please let us know the expected resubmission date by replying to this email.

When you are ready to resubmit, please upload the following:

[1] A letter containing a detailed list of your responses to all review comments, and a description of the changes you have made in the manuscript. Please note while forming your response, if your article is accepted, you may have the opportunity to make the peer review history publicly available. The record will include editor decision letters

(with reviews) and your responses to reviewer comments. If eligible, we will contact you to opt in or out

[2] Two versions of the revised manuscript: one with either highlights or tracked changes denoting where the text has been changed; the other a clean version (uploaded as the manuscript file).

Important additional instructions are given below your reviewer comments.

Thank you again for your submission to our journal. We hope that our editorial process has been constructive so far, and we welcome your feedback at any time. Please don't hesitate to contact us if you have any questions or comments.

Sincerely,

Joachim Morschhäuser

Academic Editor

PLOS Pathogens

Alex Andrianopoulos

Section Editor

PLOS Pathogens

Kasturi Haldar

Editor-in-Chief

PLOS Pathogens

orcid.org/0000-0001-5065-158X

Michael Malim

Editor-in-Chief

PLOS Pathogens

orcid.org/0000-0002-7699-2064

***********************

Reviewer Comments (if any, and for reference):

Reviewer's Responses to Questions

Part I - Summary

Please use this section to discuss strengths/weaknesses of study, novelty/significance, general execution and scholarship.

Reviewer #1: The manuscript by Cravener et al. investigates the regulation of filamentation and biofilm formation by C. albicans, and focuses on the fact that different clinical isolates show differences in these phenotypes yet there is a shared transcriptional response mediated by Efg1.

The work is interesting, of high quality, and leads to the identification of WOR3 as a novel regulator of biofilm formation, in addition to its established role in regulating white-opaque switching and gastrointestinal colonization. Several comments/suggestions are listed below on potential edits to further improve the paper.

Many thanks for this supportive summary!

Reviewer #2: In this study the Mitchell lab investigate the network of genes involved in C. albicans biofilm formation through the analysis of a large number (17) distinct strains, primarily clinical, with the goal of establishing what elements and connections are generically critical for the process, and which are strain dependent. This builds on previous studies that assessed a smaller number of strains.

The initial experiments involve constructing and characterizing efg1 nulls in a set of 16 sequenced clinical isolates as well as the general lab standard strain Sc5314. Although there were wide differences in hyphal formation and biofilm formation in the various strains, the efg1 nulls were uniformly poor at both making hyphae and making biofilms, independent of the capacity of the initial strain. Next RNA-seq was applied to all the WT/egf1 mutant strain pairs to establish the genes that were up or down regulated and determine if there is a core set of Efg1 regulated genes. 97 genes were established as Efg1 activated, while 13 were consistently found to be Efg1 repressed. The Efg1 up-regulated genes were GO term enriched for biofilm formation, as well as for genes

that had promoters found to be bound by Efg1 in other studies. Somewhat surprisingly, we are not given any general assessment of the apparently Efg1 repressed genes.

Many of the core genes represent classic hyphal genes; this is good because it connects the work to previous studies, but at the same time it challenges the need for this study if it is primarily confirming our current understanding of the circuitry. By connecting the expression profiles with hyphal development to identify correlated genes the manuscript expands the “regulatory network” to 150 genes and identifies a number of genes whose functions are not well understood, even though they have previously been noted to have enhanced expression in hyphal cells.

In response to these comments, we have included a brief summary of Efg1-repressed genes in the revised lines 133-143.

The next line of research branches out from the standard picture, investigating genes that are Efg1- dependent, but not Efg1 direct targets. Because they are part of the core they are likely to have universal rather than strain-specific regulation. The obvious candidates to provide Efg1- dependence without Efg1 binding would be Efg1-dependent and bound transcription factors; these were noted to be Brg1, Orf19.6888, Rfx2 and Ume6, with Lys143, Tye7 and Wor3 just missing the cut-off for universality. These TFs were tested as candidates for regulating the core Efg1 targets that were not directly bound by Efg1 by up-regulating their expression and making it independent

of Efg1 by a promoter switch; this was done for 3 of the 17 strains under study. This analysis showed little effect on core gene transcription for Lys143, Tye7, Rfx2, and Orf19.6888, while Brg1 and Ume6 activation generated up-regulation of many core genes. The effected gene include both direct Efg1 targets and indirect targets, and thus their relationship to Efg1-mediated transcription regulation is complex – Ume6 and Brg1 have already been extensively investigated in terms of hyphal development and biofilms. Of more novelty is the situation with Wor3, which also serves to

bypass the efg1 block for many direct and indirect Efg1 targets. Wor3 has not been linked to biofilms, but is connected to Efg1 in the circuit controlling the white/opaque switch.

Because Wor3 opposes Efg1 in the white/opaque switch, but seems to support it in biofilm formation, the role of Wor3 is looked at more closely. Here only 2 strains are examined for the effect of a wor3 deletion on biofilm formation; biofilm formation was blocked in both in assays on RPMI+FBS media, and catheter biofilm formation was reduced in vivo. Finally, the manuscript assesses whether Wor3 involvement in biofilm formation involves Brg1; activation of Wor1 can stimulate biofilms even in a brg1 mutant.

This paper has much to offer. It provides an impressive collection of gene expression profiles influenced by Efg1 across many strains, providing a clear picture of the consistencies and variations in that regulation circuit. It identifies a new relationship between Efg1 and Wor3 in the formation of hyphae and biofilms, and it sells the point that circuitry investigated only in the “model” strain can miss important layers of regulation, and provides support for the idea that assessing 3 to 4 distinct strain backgrounds will capture the bulk of the variation and allow distinction between generic and strain specific processes.

Many thanks for highlighting the positive features of this study!

Reviewer #3: A complex regulatory network regulating biofilm formation, itself a complex developmental event, has been defined in the predominant laboratory strain of Candida albicans, SC5314. Work from the Mitchell lab and others has suggested that reliance on a single strain to understand such networks may be unwise and here they expand the analysis of a core transcriptional regulator, Efg1, to 12 additional clinical isolates, up from five in earlier publications.

They find a fair bit of noise in the Efg1 regulons (or perhaps not noise, but interstrain variations whose significance is unclear), but also a core set of ~110 genes, some of which are likely indirect. Also in common is that deletion of EFG1 greatly reduces hyphal growth and biofilm formation in all 17 strains.

Of the core genes several are transcription factors, and three seem to have significant interactions with Efg1. Brg1 and Ume6 have previously been implicated in both interactions with Efg1 and biofilm formation; Wor3 had not. All three genes significantly rescued biofilm formation in an efg1Δ mutant when overexpressed.

There are important lessons in this paper about reliance on a single strain to define expectations, which the Candida field has done for far too long. That said, this is not a new revelation and so this feels slightly incremental, especially with another paper from the same group that asked similar questions in five strains. They demonstrate here that going from five to 17 strains perhaps does not change the story that much (Fig. 10). To be fair, they had to do the experiment with all 17 strains to show they probably didn’t need to do 17 strains.

However, this represents a fair bit of work, reinforces that there are important differences between strains, identifies a new role for Wor3 (and one contrary to expectations), and expands our understanding to phenotypic and regulatory differences between strains. The subject matter is appropriate for PLoS Pathogens. There are a few concerns, however.

Thank you for this positive summary. The point about the work seeming incremental caused us to revise in a few ways. Most importantly, we have tried to streamline the presentation, especially with regard to things that were known or readily deduced from previous work. In addition, we have tried to spell out what we see as the major contributions here at the end of the “Architecture” section (lines 339-353) and in a “Concluding remarks” section at the end of the Discussion (lines 387-401). I hope that these discussions are useful and on point.

Part II – Major Issues: Key Experiments Required for Acceptance

Please use this section to detail the key new experiments or modifications of existing experiments that should be absolutely required to validate study conclusions.

Generally, there should be no more than 3 such required experiments or major modifications for a "Major Revision" recommendation. If more than 3 experiments are necessary to validate the study conclusions, then you are encouraged to recommend "Reject".

Reviewer #1: I did not see major experiments required, except perhaps for more details on the physical properties of cells and biofilms under conditions where target genes are overexpressed under the TDH3 promoter (see below).

Yes, we have included some pretty interesting confocal images in Figures 7 and S8.

Reviewer #2: In the end the most solidly supported take-home message is that Wor3, like Wor1, seems to have a positive relationship with Efg1 in biofilm formation, in contrast to the negative relationship in white/opaque switching. How this “switch” occurs is not established. The title suggests that we are getting a central understanding of genetic “buffering”, but this is primarily based on speculation, and is not rigorously tested in the study. No follow-up on the repressed core genes is done – are they part of a common process, and how a TF switches from activating to repressing in this context is not discussed.

We are actively studying the mechanism that alters Wor3 activity, but those studies are at a preliminary stage right now. However, we offer some speculation that is grounded in the literature in lines 377-384. We speculate that the heat shock response, which is known to govern biofilm/hyphal genes, is responsible for modifying Wor3 activity.

We have included a brief discussion of the Efg1-repressed genes in lines 133-143. They are an interesting topic for future study, but we chose to focus on Efg1-activated genes because they were clearly connected to biofilm formation. We did get *WOR3* out of it, so our efforts were not in vain!

I was not quite sure if you thought we should change our title – I am happy to do so. But let me explain my perspective on the genetic buffering issue, which you say is based on speculation. I would say that it is based on analyzing 17 strain pair RNA-Seq datasets. The strains we chose are diverse, based on their genome sequences and phenotypic behavior documented by others, and by the fact that most Efg1-responsive genes are limited to only one or a few strains. So when we see a subset of Efg1-responsive genes that are common to all strains, it suggests that there is a mechanism to accomplish conservation of that part of the response. We actually did experimentally test whether the trio of Wor3, Brg1, and Ume6 are reasonable candidates that mediate this conservation – by overexpressing them in *efg1*Δ/Δ mutants and assaying not just cell phenotype but gene expression output. The fact that we did this in three diverse strains and got similar outcomes supports the idea that they mediate buffering, because their impact on multiple targets is uniform. Does this make sense? Or did I misunderstand your comment?

Reviewer #3:

a. The interstrain variation in the Efg1 regulons is large, several times larger than the core response. The cut-offs used for significant expression differences, however, are small, log(2)>1, p<0.05. In our experience there is some noise in these experiments and I question whether these cut-offs might artificially inflate the number of Efg10-regulated genes. By my count there are 775 Efg1-regulated genes in SC5314, of which 440 (57%) are between [log(2)] 1-2. I suspect the number might be similar with other strains. I’d encourage the authors to consider whether more stringent cut-offs might bring these regulons more in line with each other.

This is a great point! We modified Figure 10 to include a panel B with a table of mean numbers of Efg1-responsive genes with fold changes of 1 log_2_, 2 log_2_, or 3 log_2_. It’s interesting that regardless of the cutoff, about 10-15% of the Efg1-regulated genes in any one strain are common targets in all 17 strains.

b. There is also a limited discussion of the differences between the well-defined and more complex regulatory network defined in several labs (including these authors) and the four TFs the focus on here. Is the network fundamentally different from strain to strain? How does the model in Fig. 11 fit with the network defined in various papers from the Mitchell, Johnson, and Nobile labs?

For Efg1, our results indicate that yes, the network is quite different from strain to strain. This specific conclusion seems pretty clear. To address the issue of network differences among strains, we have added a more explicit paragraph, lines 285-297, that begins, “How does such extensive strain variation among Efg1-responsive genes arise?” There is also a related subsequent paragraph, lines 299-304, that begins, “Efg1 network variation may contribute to phenotypic variation.” Finally, I tried to capture the essence of our findings in the “Concluding remarks” section that includes this text (lines 392-401):

“Does our study change the broader understanding of the *C. albicans* biofilm regulatory network [9]? Our results emphasize the issue that biofilm formation and its control are condition-dependent [29]. This point has been acknowledged previously in the context of environmental variation [6, 9], but to our knowledge there was limited appreciation of the overarching impact of *C. albicans* strain variation on biofilm circuitry prior to the present study and our two related reports [11, 15]. Definition of the constant features of biofilm formation among diverse *C. albicans* isolates offers a simple rationale to prioritize circuits and targets for in depth analysis. Finally, we note that similar approaches to ours could be applied to gain better understanding of any regulatory network, and to discover new regulators that direct network behavior, including features that differ among strains and features common to all strains.”

Do you think that this is adequate? Suitable?

The detailed network model developed by the Nobile and Johnson labs is extremely valuable for identifying key target genes and understanding TF interactions. What our results suggest is that some details of the model have limited applicability – which is probably true for every model of everything! That is why I said above that our work helps to prioritize what is generally applicable.

c. The assays used are restricted to RPMI+serum (and YPD+serum in one case), a very potent biofilm-inducing condition and wor3 mutants have significant phenotype. Yet the reduction in catheter biomass is modest (if significant). This highlights differences in mutant phenotypes in different conditions and without testing these mutants (or the overexpression strains) in more conditions, they should probably temper their conclusions about the broad applicability of their network model.

Good point. To recognize the limitations of our work, I added to the Fig. 11 legend (lines 782-783), “Network relationships were deduced from one set of growth conditions and three diverse *C. albicans* strain backgrounds.” We did mention previously (lines 238-237), “Our results indicate that *WOR3* has a positive role in biofilm formation in vitro, and that its impact depends upon growth conditions.” Then where we refer to Fig. 11 in the text (lines 333-334), I qualified the conclusion with, “…Brg1 and Wor3 are the major intermediaries that link Efg1 activity to expression of Efg1 indirect targets under our growth conditions.” And in the Discussion (lines 360-361) we now say, “However, *WOR3* is not required for biofilm formation under all growth conditions.”

Part III – Minor Issues: Editorial and Data Presentation Modifications

Please use this section for editorial suggestions as well as relatively minor modifications of existing data that would enhance clarity.

Reviewer #1:

1. There is no author summary as is normal for PLos Pathogens?

Thanks for catching this. I added a summary in lines 41-50.

2. I was confused about the big picture logic statements in some places. The authors investigate why certain clinical isolates show differences in their biofilm/filamentation phenotypes, and yet also focus on the shared Efg1 expression program across isolates. Ultimately, they identify downstream targets of Efg1 that are transcription factors (like WOR3) and show that these factors can also promote biofilm formation even in the absence of Efg1 when ectopically expressed. I realize the publication by Do et al. focuses more on what differences are responsible for strain-to-strain

variation, but discussing what is the same vs. what is different would help the reader. Ultimately, does the current study also provide insight into strain-specific differences?

We have expanded our discussion of strain differences in a few ways, though we kept this material succinct because it was not a focus of this particular study.

- lines 85-94. We include a summary of how partner TFs shape Efg1 outputs (a recap of Do et al. 2022).
- lines 285-297. We provided simple instructions for how an enterprising investigator might use our data to dissect strain differences in Efg1 outputs.
- lines 299-304. We provided a simple explanation for the relationship of Wor3, Brg1, and Ume6 to differences in hypha-associated gene expression among strains.

Are WOR3 levels different between strains and contributing to phenotypic differences?

On this note, the abstract says that “Positive control of Efg1 direct target genes by other Efg1 direct target genes – BRG1, UME6, and WOR3 – may buffer core Efg1-responsive gene expression against the impact of genetic variation in the C. albicans species.” But is this true? Or could variation in expression of BRG1, UME6 and WOR3 be contributing to phenotypic variation?

The idea that Brg1/Ume6/Wor3 may contribute to phenotypic variation is an excellent point. As noted immediately above, we now include in lines 299-304 a simple explanation for the relationship of Wor3, Brg1, and Ume6 to differences in hypha-associated gene expression among strains.

In terms of buffering, let me repeat the logic I laid out above for Rev 2 and see what you think. Our RNA-Seq data indicate that most Efg1-responsive genes are limited to only one or a few strains. So when we see a subset of Efg1-responsive genes that are common to all strains, it suggests that there is a mechanism to accomplish conservation of that part of the response. We experimentally tested whether the trio of Wor3, Brg1, and Ume6 are reasonable candidates that mediate this conservation – by overexpressing them in *efg1*Δ/Δ mutants and assaying not just cell phenotype but gene expression output. The fact that we did this in three diverse strains and got similar outcomes supports the idea that they mediate buffering, because their impact on multiple targets is uniform. I laid out the idea more explicitly in the “Architecture” section, emphasizing the significance in lines 339-353. Does this make sense? Or did I not understand the criticism?

Furthermore, I would also suggest incorporating the work from the Krysan lab that describes the biofilm network as a small world network (with high fragility).

We have cited the fragile network paper from Krysan in our “Concluding remarks” for their explanation that biofilm formation requires specific environmental conditions. However, the “fragile network” concept is highly medium dependent. In that article, Glazier and Krysan refer to two specific studies in support of network fragility, their own from 2017 and Nobile from 2012. Those studies used YETS and Spider medium, respectively. There’s nothing wrong with that, but those are conditions in which each “master” TF is essential for biofilm formation – which leads to the conclusion that the network is fragile, of course. If you look at Hirakawa 2015, most of the strains that we use do not produce hyphae efficiently in Spider. That is why we use RPMI + serum. (We have not tested YETS with these strains but we have had other problems with that medium, a discussion for another day.) In RPMI + serum, the network is not that fragile. For example, Huang 2019 showed that Bcr1 and Ume6 are dispensable for biofilm formation in some strains. The whole growth medium issue is phenomenological at this stage, hence not terrifically interesting. It could be informative in the future. In any case, I hope to avoid discussing network fragility because it gets into this growth medium issue.

3. Line 159 - “We hypothesized that direct targets may govern expression of indirect targets”. How else would indirect targets be regulated other than via direct targets? I note that this is also the model proposed in Supplemental Figure S5 from Nobile et al, Cell, 2012, where the core network TFs activate downstream genes directly or via other TFs.

I deleted the sentence.

I believe that the concept that indirect targets are governed by direct targets was articulated by Herskowitz, 1989, “A regulatory hierarchy for cell specialization in yeast” in which the *MAT* locus regulates mating response genes indirectly via control of *STE12*, and meiotic genes indirectly via control of *RME1*. The other origin of the idea I think is from early work on λ phage, in which cI controls early genes indirectly through N, and N controls late genes indirectly through Q. I learned the phage stuff from David Botstein and a chalkboard so I do not have a precise reference. In any case, I am sure that Sandy would not claim to be the originator of the idea; he worked on λ phage and yeast cell type control.

4. On this note, it is perhaps worth mentioning that WOR3 was not picked up in the Nobile screen as it was not one of the factors included in their TF library.

I added in lines 361-362, “*WOR3* was not included in some earlier screens for biofilm [13] or adherence [28] mutants, and may have been undetected in others [29] due to the medium used for assays.

5. What is the structure of the TDH3-WOR3 (efg1 KO) biofilm given that this strain does not form hyphae and yet forms biofilms similar in mass to the wildtype control? Is this forming a robust biofilm (Fig. 7) without making normal hyphae? Or are hyphae forming under biofilm conditions but not in planktonic conditions (Fig. 6). This is hard to tell from the images in Fig. 8.

This was such a helpful comment – thank you! We have included imaging in the new Fig. 7 that shows that the TDH3-WOR3 (efg1 KO) biofilm indeed has some hyphal content! We are now looking into the genetic control of hypha formation in these biofilms.

6. It would actually be great to have some quantification of the hyphal cells (percentage/length?) in the TDH3-driven strains.

At this stage we would have to collect more images to quantify properly. I’d like to omit the quantification for that reason.

7. There appear to be (undeclared) splices in the lateral biofilm images in figures 2, 8 and 9. These should be modified to be clearer as to what is shown.

We have modified the images to remove splices. We used a dark background to reduce high-contrast eye fatigue.

Additional points

It is slightly surprising that TDH3-driven expression levels are not much higher than those observed from the natural promoter given that TDH3 is a very strong promoter?

Excellent point! When we first used the *TDH3* promoter eons ago we expected to see massive overexpression of just about any gene. That turned out not to be the case for multiple *ZAP1* targets (Nobile 2009 PMID: 19529758) or for *ACE2* and *ZAP1* (Finkel 2012 PMID: 22359502). We typically see RNA levels from *TDH3* promoter fusions to be 1x to 4x the wild type level. Actually it is better this way, because we usually employ it to “restore” expression in a mutant, rather than to overproduce something for biotechnological purposes. So yes, we share your surprise.

Line 169. “As a readout, we used Nanostring probes to measure expression of several indirect and direct targets (S6 Table).” Would be good to be precise here and to give the number of genes being analyzed by Nanostring. Looks like it is 103 genes from the table?

Done.

Would it make sense to split Figure 4 into direct and indirect targets of Efg1? Just to highlight the genes from both groups? This data is in the table but a graphical summary would give the reader a clearer picture of the pattern of regulation.

Done.

For Figure 4, I believe that all of the expression data is from strong hyphal-inducing conditions. This could be made stated in the text and in the legend.

Good point.

Legend, lines 726-727, “RNA expression levels were calculated relative to the *efg1*Δ/Δ for each *TDH3-TF* strain under strong hypha-inducing conditions (RPMI+10% FBS at 37°C).”

Text, lines 172-173, “As a readout, we used Nanostring probes to measure expression of 103 direct and indirect targets under strong hypha-inducing conditions (S6 Table).”

Figure 5 could be moved to supplemental material.

Done.

Line 322 “However, a Efg1 core17-exp direct target like HWP1 will be Efg1-responsive even if Efg1 itself cannot activate HWP1 by direct binding because of the intervention of Brg1, Wor3, and Ume6.” Suggest adding the underlined words to make clear what is meant. Throughout the text and figures (e.g. Figure 11) it could be emphasized that direct targets of Efg1 refer to direct binding of Efg1 to those promoters, as this may not always be obvious to all readers.

We deleted the specific sentence you referred to in an effort to streamline the manuscript and make room for additional text.

Direct/indirect reminders:

- Figure 4 legend (lines 725-726). “Direct targets are genes whose 5’ regions are bound by Efg1; indirect targets are genes whose 5’ regions are not detectably bound by Efg1 [11-13].”
- This is also still mentioned in the Abstract (lines 29-30). “…includes 68 direct Efg1 targets, whose 5’ regions are bound by Efg1 in five clinical isolates, and 42 indirect Efg1 targets, whose 5’ regions are not detectably bound by Efg1.”
- Lines 98-99. “Does such a principal network include only direct Efg1 target genes, that is, those genes whose 5’ regions are bound by Efg1?”
- Lines 156-160. “…68 direct Efg1 targets, which are genes whose 5’ regions are bound by Efg1, based on ChIP data [11-13]. … 42 genes are indirect Efg1 targets, which are genes whose 5’ regions are not detectably bound by Efg1 [11-13].”

Reviewer #2: (No Response)

Reviewer #3: Fig. 1 could benefit from either headings (WT/efg1Δ/Δ) or a panel A/B. Or an organization more like Fig. 2.

Done.
